# Supplementary material for: Three-dimensional visualisation of the internal anatomy of the sparrowhawk (Accipiter nisus) forelimb using contrast-enhanced micro-computed tomography
Source: PeerJ. 2017 Mar 15;5:e3039. doi: 10.7717/peerj.3039 (PMC5356476; doi:10.7717/peerj.3039)
Supplement: Supplemental Information 2 — Muscle volumes (cm3) of a sparrowhawk (Accipiter nisus) wing. Volumes obtained during gross dissection of the right wing (fresh wing) and the scanned wing after being washed, and measured from the isosurfaces of the 3D model. Abbreviations as indicated in Fig. 2. [file peerj-05-3039-s002.docx]

| **File S2.** Muscle volumes (cm^3^) of a sparrowhawk (*Accipiter nisus*) wing. Volumes obtained during gross dissection of the right wing (fresh wing) and the scanned wing after being washed, and measured from the isosurfaces of the 3D model. Abbreviations as indicated in Figure 2. | | | |
| --- | --- | --- | --- |
| **Muscle** | **Fresh wing** | **Scanned wing** | **3D model** |
| ABA | 0.017 | 0.012 | 0.019 |
| ADM | 0.048 | 0.042 | 0.023 |
| ADA | 0.014 | 0.014 | 0.021 |
| EBA | 0.006 | 0.007 | 0.006 |
| FA | 0.009 | 0.009 | 0.018 |
| FDM | 0.018 |  | 0.001 |
| ID | 0.023 | 0.030 | 0.042 |
| IV | 0.045 | 0.030 | 0.024 |
| UD | 0.085 | 0.108 | 0.102 |
| ECTU | 0.137 | 0.121 | 0.139 |
| ECR | 0.775 | 0.515 | 0.461 |
| ECU | 0.205 | 0.395 | 0.137 |
| EDC | 0.234 | 0.172 | 0.154 |
| ELA | 0.115 | 0.097 | 0.096 |
| ELDM | 0.054 | 0.076 | 0.039 |
| FCU | 0.937 | 0.601 | 0.578 |
| FDP | 0.137 | 0.087 | 0.107 |
| FDS | 0.175 | 0.081 | 0.074 |
| PP | 0.539 | 0.292 | 0.319 |
| PS | 0.191 | 0.113 | 0.125 |
| SU | 0.079 | 0.020 | 0.063 |
| UV | 0.089 | 0.063 | 0.067 |
| BB | 1.328 | 0.954 | 0.754 |
| BR | 0.053 | 0.034 | 0.047 |
| CCR | 0.022 |  | 0.055 |
| DMA | 1.298 | 1.070 | 0.947 |
| DMI | 0.075 | 0.054 | 0.022 |
| HT | 1.092 | 0.862 | 0.839 |
| ST | 0.443 | 0.315 | 0.274 |
| TP | 0.705 | 0.392 | 0.311 |
